# Pulsed laser synthesis of highly active Ag–Rh and Ag–Pt antenna–reactor-type plasmonic catalysts

**DOI:** 10.3762/bjnano.10.192

**Published:** 2019-09-26

**Authors:** Kenneth A Kane, Massimo F Bertino

**Affiliations:** 1 Department of Physics, Virginia Commonwealth University, Richmond, Virginia, 23220, USA

**Keywords:** Ag, antenna–reactor, catalysis, heterostructures, laser ablation, multicomponent, nanoparticles, 4-nitrophenol, plasmonic, Pt, Rh

## Abstract

Ag, Pt, and Rh monometallic colloids were produced via laser ablation. Separate Ag–Rh and Ag–Pt heterostructures were formed by mixing and resulted in groupings of Rh/Pt nanoparticles adsorbing to the concavities of the larger Ag nanostructures. The 400 nm Ag plasmonic absorption peak was slightly blue-shifted for Ag–Pt and red-shifted for Ag–Rh heterostructures. Catalytic activity for the reduction of 4-nitrophenol increased significantly for Ag–Pt and Ag–Rh compared to the monometallic constituents, and persisted at lower loading ratios and consecutive reduction cycles. The enhancement is attributed to the Rh and Pt nanoparticles forming antenna–reactor-type plasmonic catalysts with the Ag nanostructures.

## Introduction

Metal nanoparticles can interact with visible light through an excitation of the localized surface plasmon resonance (LSPR). The LSPR is a resonant, collective oscillation of the free electrons of the metal that occurs when the dielectric constants of the metal and the medium (through which the free electrons oscillate) are appropriately matched and the wavelength of the incident light is longer than the size of the nanoparticle (NP). A consequence of plasmon excitation is the direct confinement of light near the surface of the metal NP in the form of elevated electric fields [1]. The confinement amplifies absorption (electron–hole pair excitation) and photon scattering, both of which are photophysical processes [2–5]. For isolated NPs, the amplification can be as high as hundreds of times the intensity of the incoming electromagnetic field [6] and thousands of times the intensity at corners or other sharp features of the NP [7].

Elevated LSPR fields dissipate energy through radiative photon scattering or nonradiative absorption [8–10]. Nonradiative absorption results in the generation of energetic charge carriers [11–12] and chemical bonds can be activated with the energy created by the charge carriers, documented by Christopher et al. who reported partial oxidation reactions on plasmonic Ag NPs induced by resonant light of relatively low intensity [13]. Energy is transferred to reactant molecules on the surface in the form of hot charge or an electronic excitation and forces the atoms of molecules to reconfigure along an excited potential energy surface. The molecular reconfiguration can lead to a chemical transformation or the molecule can return to ground-state potential energy with additional vibrational energy. The process does not require charge extraction from the metal. Rather, it leads to an electronic excitation in the adsorbate–metal complex, forming transient adsorbate ions. The adsorbate ions can survive on metal surfaces tens of femtoseconds before relaxation, which is sufficient for chemical transformation or additional vibrational energy to be transferred to the adsorbate, leading to reaction [14].

There are two mechanisms that can lead to the electronic excitation in the adsorbate–metal complex. The first is the indirect transfer of energy where photoexcitation, or absorption, results in the generation of charge carriers that then collide with other charge carriers. The collisions create an energized, or hot, Fermi–Dirac distribution. From the distribution, charge carriers can then transfer from the metal to unpopulated adsorbate states. The second mechanism is the direct transfer of energy, or the direct excitation of charge carriers from the metal to unpopulated adsorbate states within the metal–reactant complex [15].

Light energy, localized at the surface of a plasmonic NP, can be transferred to a neighboring catalytic material via LSPR-induced electric fields [16–17]. Catalytic materials, such as Pt and Pd, possess an electronic structure that features d-bands at the Fermi level, which allow for direct momentum-conserved interband excitations in the visible range. The interband excitations allow catalytic materials to act as absorption sinks for the electromagnetic energy concentrated by plasmonic metals [14]. Aslam et al. demonstrated this in a system consisting of large, Ag nanocube cores (75 nm) surrounded by thin (1 nm) Pt shells [18]. Photon scattering was optically determined to be the primary means of plasmon decay for monometallic Ag. However, after Pt deposition the primary pathway for plasmon decay changed to absorption, indicating the thin Pt layer provided an alternate pathway for the dissipation of energy. Combined with electrodynamic simulations of spatial distributions of LSPR energy dissipation, the experiments concluded that charge carriers produced in the Pt shell via LSPR excitation from the Ag nanocube core could be utilized for surface chemistry. Similarly, Zhang et al. coined the term “antenna–reactor” photocatalysis by fabricating Al, surrounded by a thin layer of Al_2_O_3_, as a plasmonic metal (antenna) and Pd NPs as catalyst (reactor), where Al_2_O_3_ prevented the contact between Ag and Pd [19]. After observing an enhanced reduction of acetylene and dissociation of H_2_ via photocatalysis, it was concluded that a plasmonic antenna can focus light onto the catalytic reactor and induce a “forced plasmon” that efficiently generates hot charge carriers, transforming the catalytic NP into a photocatalytic NP.

Here, the facile synthesis of highly active Ag–Rh and Ag–Pt heterostructures for the reduction of 4-nitrophenol through pulsed laser ablation is reported. The synthesis method for the monometallic precursor colloids, which are mixed together without any post laser treatment to form unalloyed heterostructures, is based on a previously reported method [20]. The reduction of 4-nitrophenol is a widely utilized benchmark method for gauging catalytic activity [21–22] as the rate of the reduction can be directly observed via UV–vis absorption spectroscopy. Ag–Rh/Pt heterostructures exhibited a significantly increased catalytic activity compared to the constituents. The observed increase is attributed to the heterostructures forming antenna–reactor-type plasmonic catalysts, where plasmonic Ag nanostructures focus and transfer the energy of incoming photons to neighboring Rh or Pt NPs via LSPR. The attribution is based on red-shifts and blue-shifts in the Ag plasmon resonance and on reports in the literature, specifically, the previously mentioned reports of Aslam et al. [18] and Zhang and co-workers [19]. The aim of the present study is to lay the foundation for future work and illuminate the potential efficacy of pulsed laser ablation as an effective method for the production of multicomponent plasmonic catalysts. The reported method possesses several inherent benefits: (1) the absence of surfactants and ligands; (2) a simple, robust, and quick two-step method production; (3) bulk targets of the constituent metals as the only necessary ingredients.

## Experimental

The synthesis of the monometallic colloids was carried out using a EKSPLA SL312G/SH/TH pulsed laser with a pulse width of 700 ps, at the first harmonic wavelength of 1064 nm, operating at a repetition rate of 10 Hz, at a fluence of 1.5 × 10^6^ J/m^2^. The laser beam was focused, with a 10 cm focal length lens, through the side of a 2.54 cm scintillation vial and incident on Ag/Pt/Rh targets (obtained from Alfa Aesar, purity > 99.9%) in 2.0 mL of deionized H_2_O, traversing approximately 1.25 cm of solution. The mass of ablated material produced by 5 min of ablation was determined to be 100 ± 10 μg for Ag, Pt, and Rh. The Ag–Rh and Ag–Pt colloidal heterostructures were made by mixing the monometallic colloids via rapid pipette intake/release. The volume loading ratio for all Ag–Rh and Ag–Pt colloids was 1:1 unless otherwise noted. The synthesis is based on a previously reported method [20]. The key difference is that no pulsed laser treatment was performed on mixtures of monometallic colloids, eliminating any potential changes in catalytic activity due to alloying in the Ag–Pt and Ag–Rh bimetallic systems.

UV–vis absorption spectroscopy measurements were made using an OceanOptics USB200 UV–vis spectrometer. Bright-field images of NPs were obtained with a Zeiss Libra transmission electron microscope (TEM) operating at 120 kV. High-resolution bright-field imaging was carried out with a FEI Titan TEM operating at 300 kV.

Catalytic activity was obtained in the following manner: To 1.0 mL distilled water, 5 μL of fresh 10^−2^ M 4-nitrophenol solution and 0.5 mL of 50 μg/mL catalyst suspension was added. Afterwards, 0.015 mL of fresh aqueous 10^−1^ M NaBH_4_ was spiked into the mixture, as reducing agent, and the time-dependent absorption spectrum was recorded. As NaBH_4_ was used in excess, the reaction kinetics are assumed to follow a first-order rate law. The absorption peak at 400 nm (Abs_400nm_) forms immediately after the introduction of NaBH_4_, signaling the formation of 4-nitrophenolate. The intensity is proportional to the concentration of 4-nitrophenolate in solution; the peak drops as the reduction of 4-nitrophenolate proceeds. The first-order reaction rate, *k*, was estimated from the experimental data of *C*/*C*_0_ as a function of the time. The data was fitted with the function ln(*C*/*C*_0_) = −*kt*. Figure S1 (Supporting Information File 1) shows a typical absorption spectrum for the reduction over a Ag–Pt sample. Cycling experiments were performed by additional 5 μL 10^−2^ M 4-nitrophenol spikes, with every other spike accompanied by the addition of 0.015 mL 10^−1^ M NaBH_4_.

## Results and Discussion

**Figure 1:**
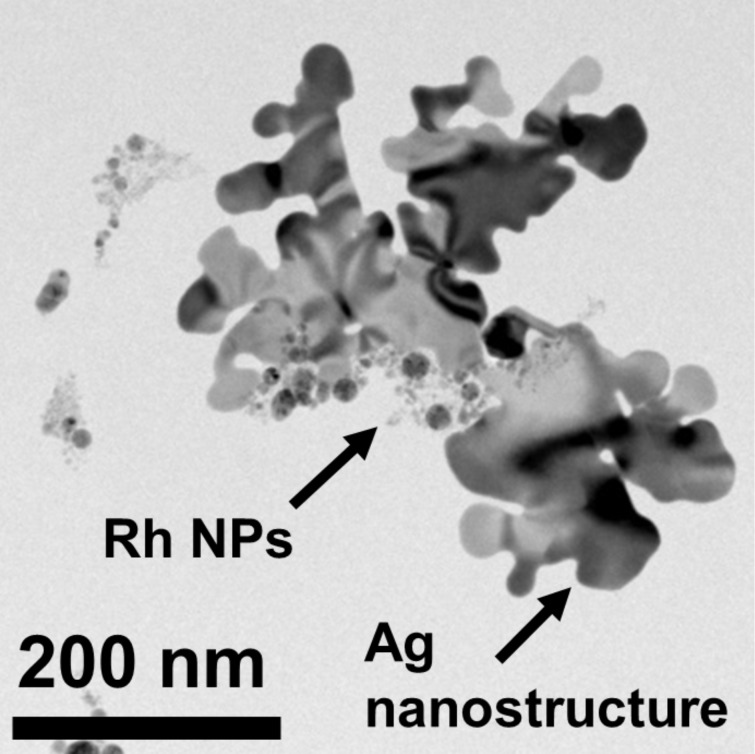
Ag–Rh heterostructures formed after mixing colloidal Ag and Rh suspensions generated via pulsed laser ablation. Rh nanoparticle groupings were observed to only adsorb to Ag nanostructure concavities. Similar Ag–Pt heterostructures formed in the mixtures of colloidal Ag and Pt suspensions.

Pulsed laser ablation generated different particle morphologies for Rh/Pt and Ag. The latter formed asymmetrical nanostructures larger than 100 nm, while the former yielded groupings of NPs with diameters below 20 nm. TEM micrographs of the pure metal samples are presented in Figure S2 (Supporting Information File 1 [20]). The combination of the Rh and Ag colloids at a 1:1 loading ratio resulted in Rh NP groupings adsorbing to Ag nanostructures, as seen in Figure 1, forming heterostructures. The NP groupings were found to only adsorb to concavities. The 1:1 Ag–Pt system formed similar heterostructures. A series of HR-TEM images confirming the atomic makeup of the heterostructures is reported in Figure S3 (Supporting Information File 1).

**Figure 2:**
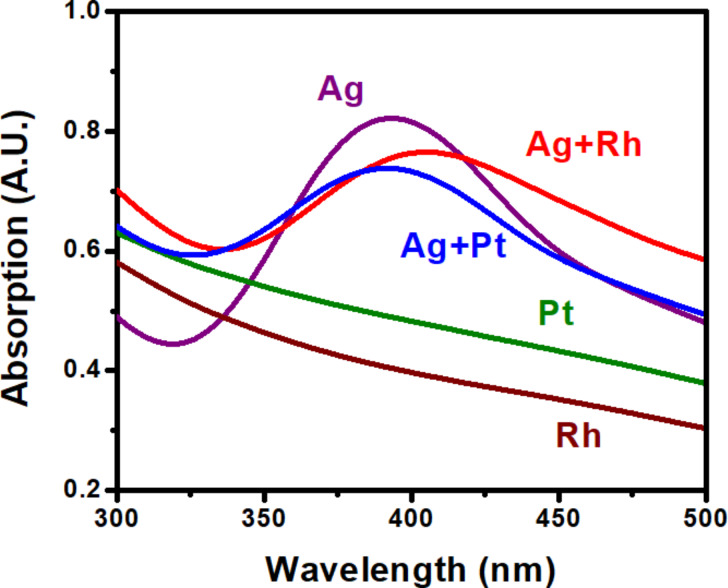
UV–vis absorption spectra of the monometallic Ag, Pt, Rh, and the colloidal Ag–Rh and Ag–Pt heterostructure solutions. Ag exhibited a plasmon absorption peak at ca. 400 nm with a slightly asymmetrical distribution towards lower energies. Ag–Rh heterostructures exhibited a red-shift with an additional asymmetrical skew towards lower energies. Ag–Pt exhibited a slight blue-shift.

UV–vis absorption spectra of the monometallic and mixed colloids are reported in Figure 2. Similar spectra were previously reported [20]. However, the spectra of the monometallic mixtures are more closely examined here. Colloidal Ag exhibited a strong plasmonic peak at ca. 400 nm with an asymmetrical distribution towards lower energies, which could be an indication of plasmonic coupling. Neighboring plasmonic NPs can couple plasmonic modes into “bonding” and “antibonding” resonances that can lead to shifts in the LSPR [1]. Asymmetrical and multifaceted structures, similar to the Ag nanostructures produced here, can also exhibit plasmonic coupling between angular features [1]. Plasmonic coupling results in an increase in the magnitude of the LSPR-induced electric fields. The selective adsorption of NP groupings to the Ag nanostructure concavities may be a result of LSPR-induced electric fields, which are largest in these highly angular regions. The mixed Ag–Pt colloid, comprising Ag–Pt heterostructures, was found to exhibit a slight blue-shift of the LSPR, while the mixed Ag–Rh colloid showed a more pronounced red-shift.

**Figure 3:**
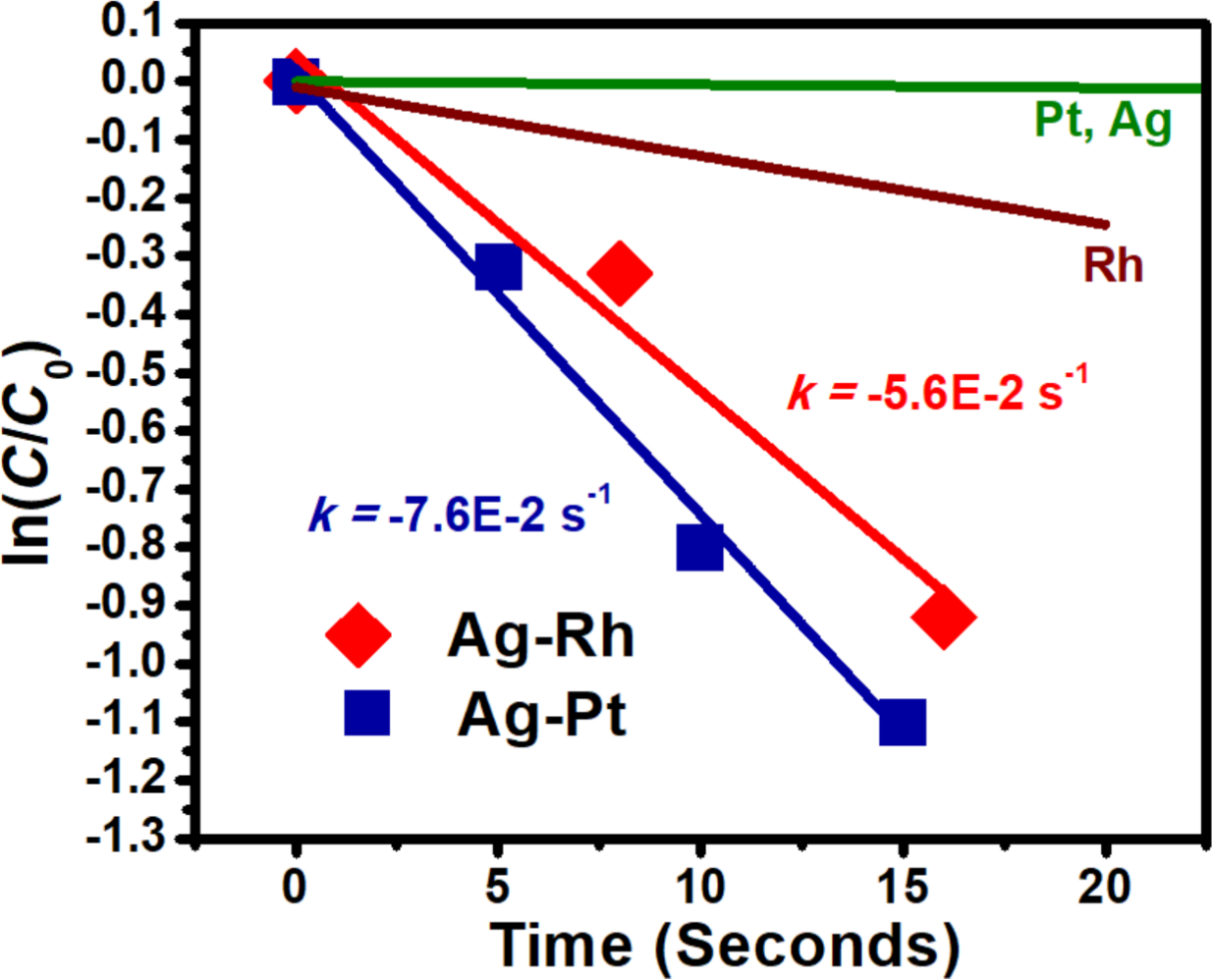
Experimentally observed rate constants in the form of ln(*C*/*C*_0_) = −*kt*. *C*_0_ is calculated from Abs_400nm_ prior to the reducing agent spike. *t* = 0 s is taken as the time the spike occurs. Lines representing the activity of monometallic Rh, Pt, and Ag are included for comparison.

Pt and Ag are known to possess lower catalytic activity in the reduction of 4-nitrophenol than Rh [21–22]. Accordingly, Pt and Ag exhibited a lower activity in this study, with rate constants of −4.0 × 10^−4^ s^−1^ and −1.1 × 10^−3^ s^−1^, respectively, while Rh had a rate constant of −1.2 × 10^−2^ s^−1^. The determination of the rate constants of the pure metals is reported in Figure S4 (Supporting Information File 1). The activity values of the Ag–Pt and Ag–Rh heterostructures are reported in Figure 3. Lines representing the activity of monometallic Rh and Pt, overlapping with Ag, are included in the figure for reference. Both heterostructure systems exhibited a significant increase in activity over the monometallic constituents, the Ag–Rh heterostructures with a rate constant of −5.7 × 10^−2^ s^-1^ and the Ag–Pt heterostructures with a rate constant of −7.6 × 10^−2^ s^−1^. It is unclear whether the monometallic Ag nanostructures contribute to the reduction photophysically, i.e., indirectly through hot charges transferring energy to the reactants or directly through excitation of Ag–reactant complexes. Regardless, the activity of monometallic Ag is low, indicating that any photophysical contribution of Ag is small. As seen above, the proximity of Rh or Pt NP groupings to Ag nanostructures results in an increase in activity. The increase is attributed to Rh/Pt and Ag forming multicomponent, antenna–reactor-type photocatalysts. Highly elevated LSPR-induced electric fields in the angular concavities increase the number of energetic charge carriers of adsorbed Rh/Pt NPs, and subsequently increase the reduction rate of 4-nitrophenolate. This effect is isolated to the NP groupings. The Rh/Pt NPs function as reactors and the Ag nanostructures serve as antenna, gathering, localizing, and transferring the energy from incoming electromagnetic fields. The modes of energetic charge carrier increase in Rh and Pt may differ, based on the differences in the Ag plasmon shift between Ag–Rh and Ag–Pt, and the differences in activity of monometallic Rh and Pt. The proximity of Rh to Ag nanostructures may result in forced plasmons at the Rh NP surfaces [19]. The forced plasmons decay via absorption, increasing the number of energetic charge carriers, and the activity of the Rh NPs is enhanced. The observable red-shift for Ag–Rh may be an indication of forced plasmon transfer, i.e., a red-shift similar to plasmonic coupling. In the Ag–Pt system, the lack of a red-shift may indicate that there is no forced plasmon transfer. Pt NPs provide an alternative mode for Ag plasmon decay and the apparent slight blue-shift for Ag–Pt indicates a shift from scattering to absorption. The Pt NPs, which exhibited low catalytic activity, are an outlet for plasmon decay and become photocatalytic. The hypothesis is based on the previously discussed findings that Ag nanocubes had a significant shift from scattering to absorption as the primary means of plasmon decay after being coated with a 1 nm Pt shell, and that the absorption was calculated to be concentrated in the Pt shell [18].

**Figure 4:**
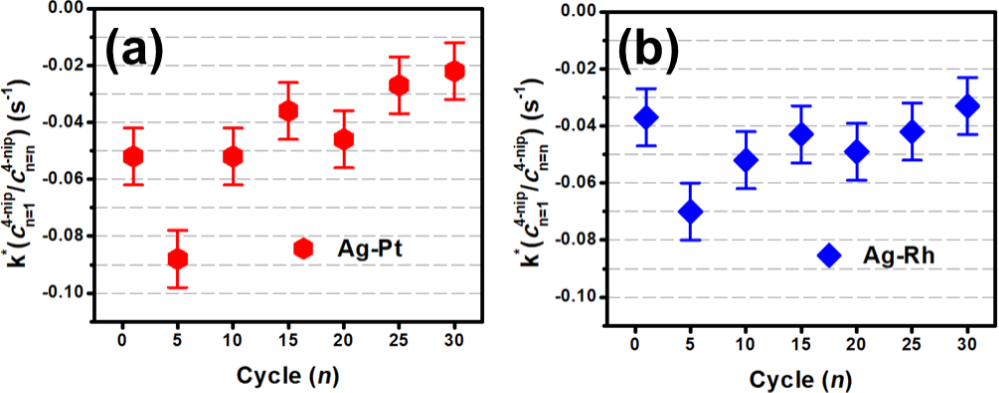
Rate constants for 1:1 loading ratios of the (a) Ag–Pt and (b) Ag–Rh systems over the course of 30 reduction cycles. The rate constant *k* is normalized for the diminishing concentration of 4-nitrophenolate over the cycling experiment. Errors bars represent the standard deviation of six separate determinations of *k* for 1:1 Ag–Rh.

**Figure 5:**
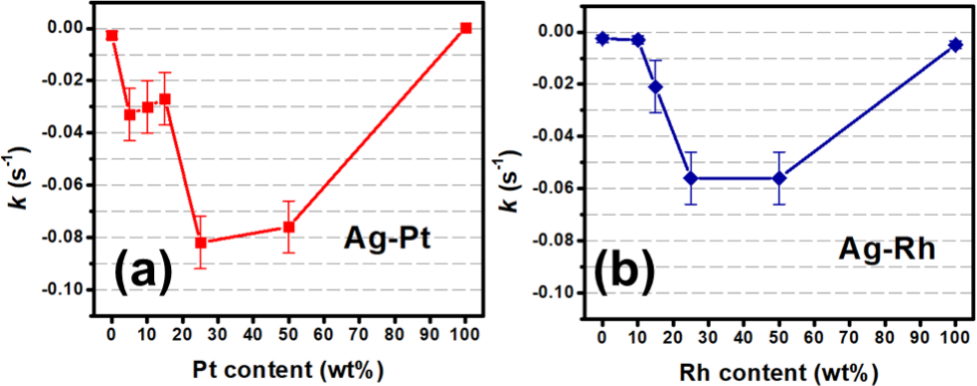
Rate constants for the (a) Ag–Pt and (b) Ag–Rh systems at a variety of loading ratios. Catalytic enhancement is observed at Pt contents as low as 5% for the Ag–Pt system and Rh contents as low as 15% for the Ag–Rh system. The rate constant *k* is normalized for the diminishing concentration of 4-nitrophenolate over the cycling experiment. Errors bars represent the standard deviation of six separate determinations of *k* for 1:1 Ag-Rh.

Figure 4a and Figure 4b show the catalytic activity of the Ag–Pt and Ag–Rh heterostructures, respectively, over the course of thirty cycles at a loading ratio of 1:1. The determined rate constant is normalized for the diminishing concentration of catalyst. The activity of Ag–Rh did not diminish over the course of thirty cycles. However, the activity of Ag–Pt did. Both systems exhibited a sharp increase in activity after five cycles that did not persist after a higher number of cycles. The exact cause is presently unclear. Due to the unorthodox nature of the cycling experiments (i.e., no washing or solvent exchange between the cycles), the results are insufficient to conclude that the heterostructures are highly cyclable. Instead, the results demonstrate that the enhancement is not a singular event and that the heterostructures are stable. In conjunction to cycling experiments, Ag–Rh/Pt loading ratio experiments were performed to gauge the magnitude of the enhancement. The activity for a series of loading ratios and the observed rate constants are reported in Figure 5a for Ag–Pt and Figure 5b for Ag–Rh. The enhancement persisted at loading ratios as low as 5% and 15%, respectively, for the Ag–Pt and Ag–Rh systems, an indication of the cost-effective potential of multicomponent plasmonic catalysts.

## Conclusion

Colloidal Ag–Rh/Pt heterostructures were obtained by mixing monometallic colloids generated using pulsed laser ablation. Both Ag–Rh and Ag–Pt colloids exhibited a shift in the Ag plasmon peak location; a slight blue-shift for Ag–Pt and a larger red-shift for Ag–Rh. The catalytic activity was measured for the reduction of 4-nitrophenol over the colloidal heterostructures and the monometallic constituents. Ag–Rh and Ag–Pt exhibited a higher catalytic activity than Ag, Rh, and Pt. The enhancement is attributed to the Ag–Pt and Ag–Rh heterostructures forming multicomponent plasmonic catalysts through an antenna–reactor-type plasmonic interaction between the Ag nanostructures and the adsorbed Rh/Pt NPs. Different mechanisms of charge carrier increase are postulated between the Ag–Rh and Ag–Pt systems based on the differences of the Ag LSPR shifts. Cycling experiments showed the heterostructures to be stable, and the activity was measured for a variety of Ag–Rh/Pt loading ratios, demonstrating that the enhancement persisted at low loading ratios. Further and future inquiry will include: (1) studying the reaction kinetics in the absence of visible light to confirm plasmonic enhancement; (2) running the reduction over the colloidal heterostructures in the absence of a reducing agent to gauge any potential Ag plasmonic contributions, and (3) exploring other antenna–reactor-type heterostructures formed through pulsed laser ablation.

## Supporting Information

File 1 Additional experimental data.
