# Supplementary material for: Pulsed laser synthesis of highly active Ag–Rh and Ag–Pt antenna–reactor-type plasmonic catalysts
Source: Beilstein J Nanotechnol. 2019 Sep 26;10:1958–63. doi: 10.3762/bjnano.10.192 (PMC6774074; doi:10.3762/bjnano.10.192)
Supplement: File 1 — Additional experimental data. [file Beilstein_J_Nanotechnol-10-1958-s001.pdf]

BEILSTEIN JOURNAL OF NANOTECHNOLOGY

Supporting Information

for

Pulsed laser synthesis of highly active Ag–Rh and Ag–Pt antenna–reactor-type plasmonic catalysts

Kenneth A. Kane and Massimo F. Bertino

Beilstein J. Nanotechnol. **2019,** *10,* 1958–1963. doi:10.3762/bjnano.10.192

Additional experimental data

License and Terms: This is a supporting information file under the terms of the Creative Commons Attribution License (http://creativecommons.org/licenses/by/4.0). Please note that the reuse, redistribution and reproduction in particular requires that the authors and source are credited.
The license is subject to the *Beilstein Journal of Nanotechnology* terms and conditions: (https://www.beilstein-journals.org/bjnano)

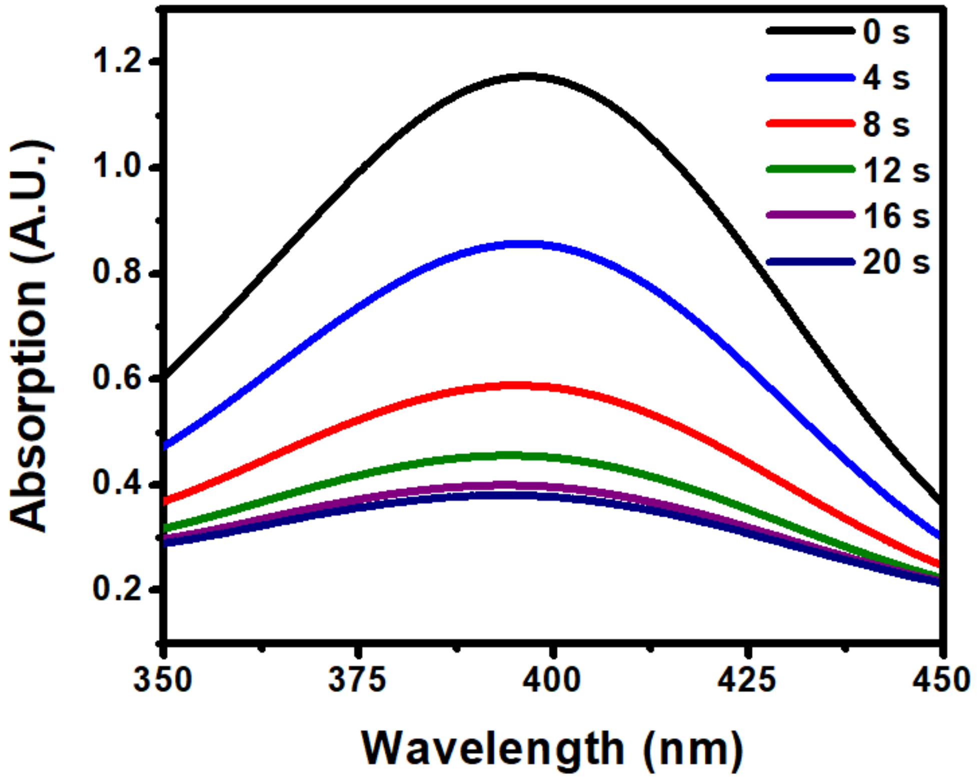

Figure S1: Time-dependent absorption spectrum of a typical 4-nitrophenol reduction over colloidal Ag–Pt heterostructures. The intensity of the absorption at 400 nm is proportional to the concentration of the 4-nitrophenolate ion in solution, which forms immediately after the introduction of $NaBH_4$.

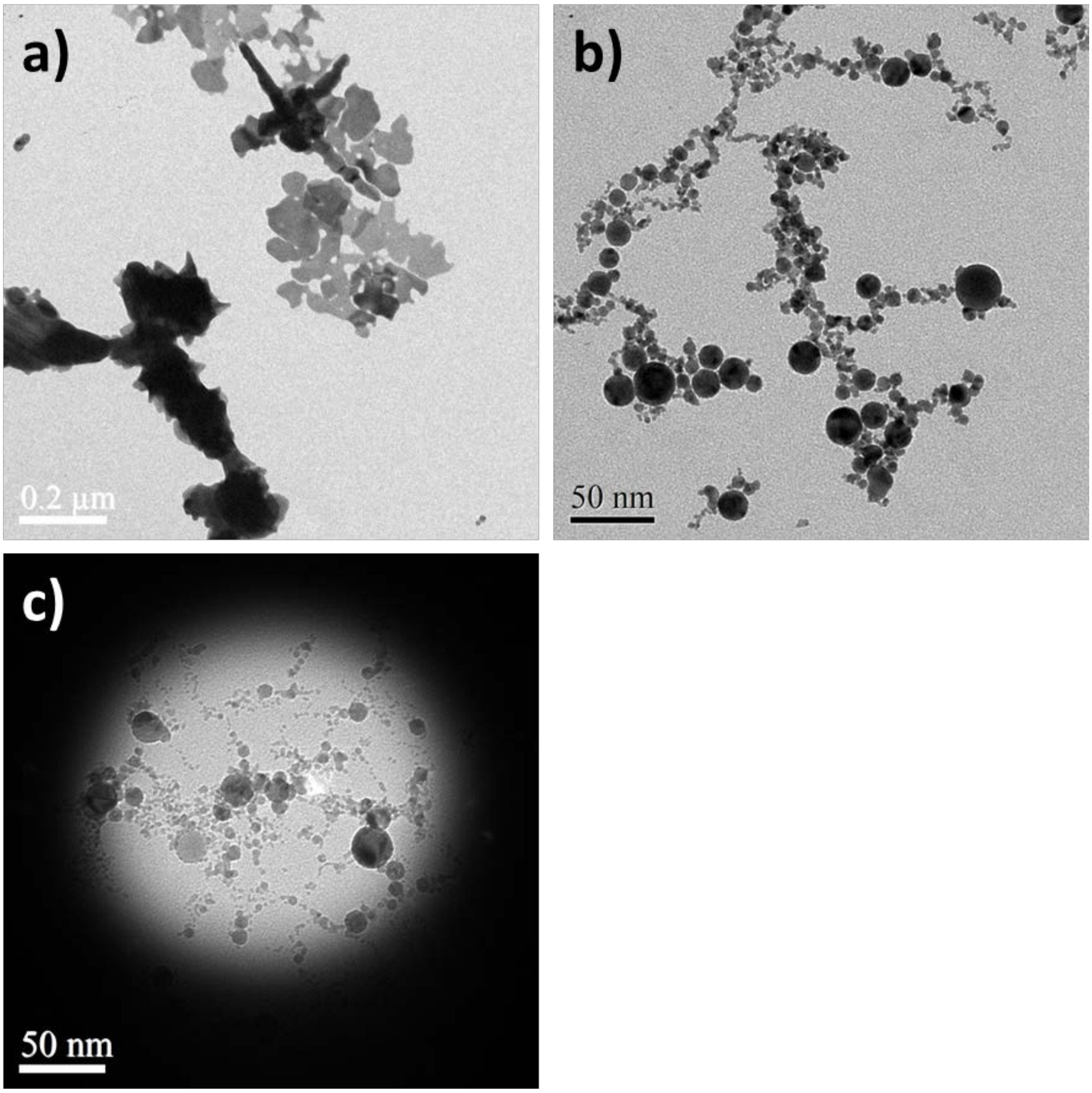

Figure S2: a) Silver nanostructures, b) platinum nanoparticles, and c) rhodium nanoparticles produced by pulse laser ablation.

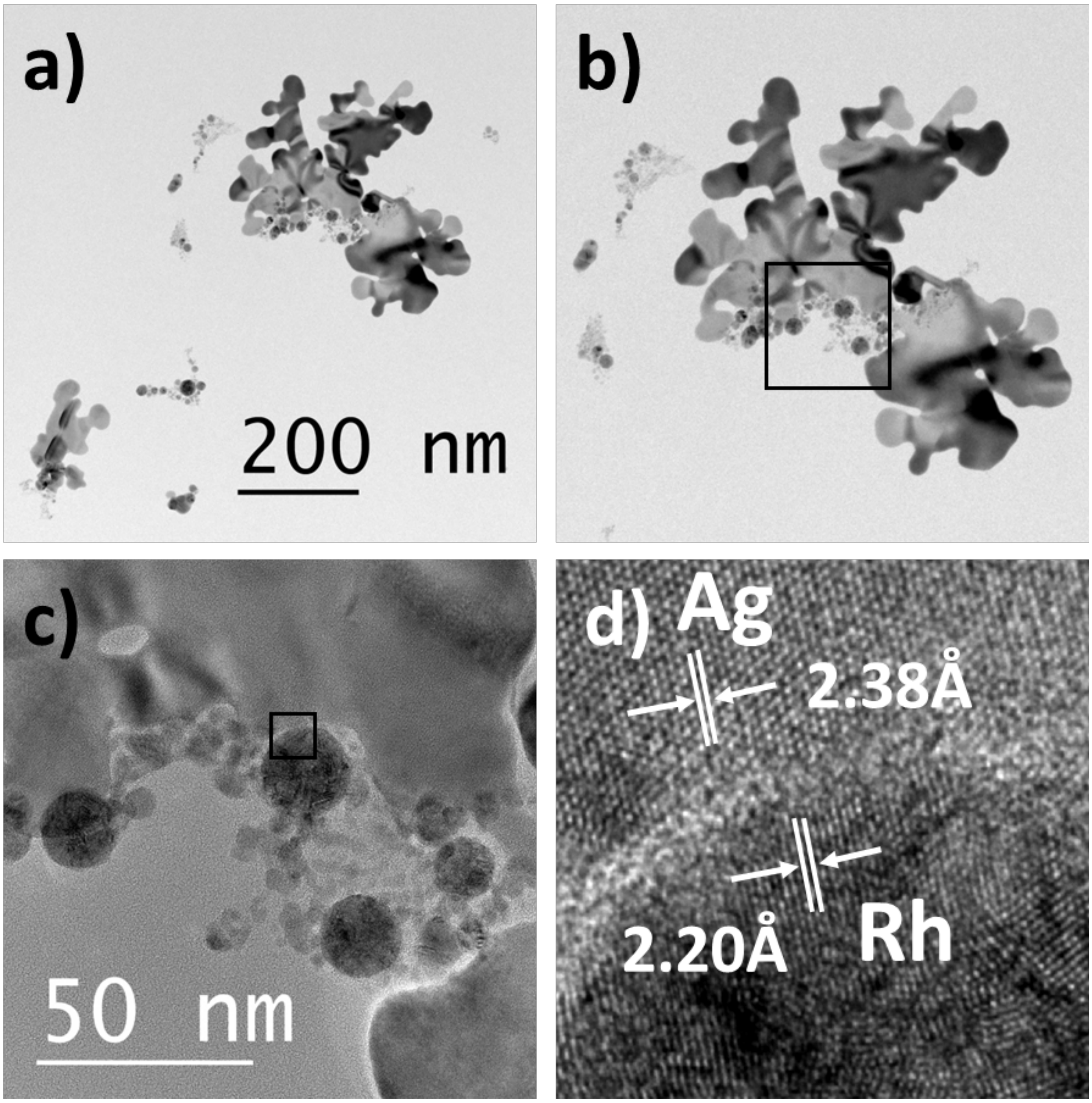

Figure S3: a) Bright-field image of mixed Ag–Rh heterostructures. b) Close up of (a) showing Rh NP grouping adsorbed onto Ag nanostructure concavity. c) HR-TEM image of highlighted area from (b) . d) Close-up of the highlighted area in (c) showing lattice spacings of Ag and Rh.

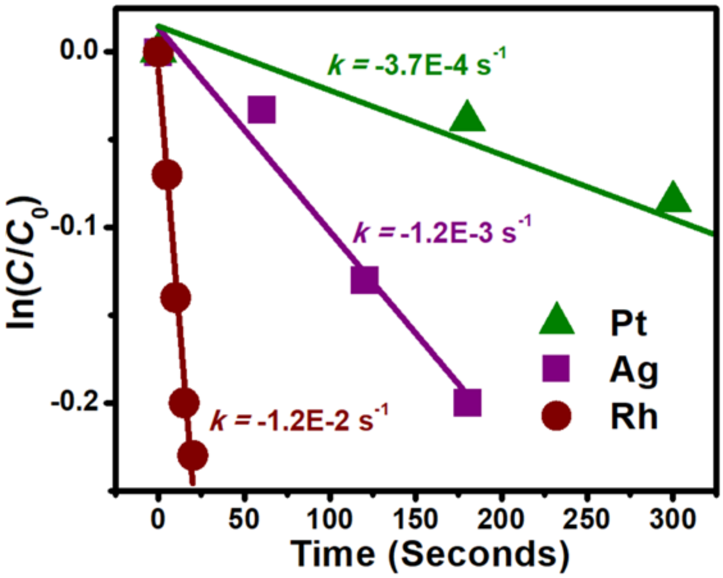

Figure S4: Experimentally observed rate constants in the form of $\ln(C/C_0) = -kt$. C_0 is taken as Abs_{400nm} prior to the reducing agent spike. t = 0 s is taken as the time the spike occurs.
